# Flotillin‐1 interacts with the serotonin transporter and modulates chronic corticosterone response

**DOI:** 10.1111/gbb.12482

**Published:** 2018-05-20

**Authors:** S. N. Reisinger, E. Kong, B. Molz, T. Humberg, S. Sideromenos, A. Cicvaric, T. Steinkellner, J.‐W. Yang, M. Cabatic, F. J. Monje, H. H. Sitte, B. J. Nichols, D. D. Pollak

**Affiliations:** ^1^ Department of Neurophysiology and Neuropharmacology Center for Physiology and Pharmacology, Medical University of Vienna Vienna Austria; ^2^ Department of Pharmacology Center for Physiology and Pharmacology, Medical University of Vienna Vienna Austria; ^3^ MRC Laboratory of Molecular Biology Cambridge UK

**Keywords:** animal model, chronic corticosterone, depression, Flotillin‐1, serotonin transporter, SERT

## Abstract

Aberrant serotonergic neurotransmission in the brain is considered at the core of the pathophysiological mechanisms involved in neuropsychiatric disorders. Gene by environment interactions contribute to the development of depression and involve modulation of the availability and functional activity of the serotonin transporter (SERT). Using behavioral and in vivo electrophysiological approaches together with biochemical, molecular‐biological and molecular imaging tools we establish Flotillin‐1 (Flot1) as a novel protein interacting with SERT and demonstrate its involvement in the response to chronic corticosterone (CORT) treatment. We show that genetic Flot1 depletion augments chronic CORT‐induced behavioral despair and describe concomitant alterations in the expression of SERT, activity of serotonergic neurons and alterations of the glucocorticoid receptor transport machinery. Hence, we propose a role for Flot1 as modulatory factor for the depressogenic consequences of chronic CORT exposure and suggest Flotillin‐1‐dependent regulation of SERT expression and activity of serotonergic neurotransmission at the core of the molecular mechanisms involved.

## 1 INTRODUCTION

Serotonin (5‐hydroxytryptamine, 5‐HT) is a neurotransmitter central to the generation and regulation of emotion and cognition. Alterations of serotonergic neurotransmission have been critically implicated in the etiology of several psychopathologies including mood and anxiety disorders and substance abuse.^1^, ^2^, ^3^, ^4^ The serotonin transporter (SERT, SLC6A4) is the major regulator of serotonergic tone in the brain through the control of the reuptake of released serotonin from the synaptic cleft into the presynaptic terminal. As a consequence, SERT directly modulates the intensity and duration of 5‐HT‐dependent signaling and regulation of SERT expression and activity. SERT has therefore been a central focus of pharmaceutical interest as a druggable target in the therapeutic management of affective disorders.^5^, ^6^, ^7^, ^8^ Additionally, genetic variations in the promoter region of the SERT gene have been proposed to be involved in mediating the gene × environment effects involved in the pathogenesis of depression.^9^, ^10^, ^11^

Nevertheless, the “serotonin hypothesis” of depression, which has not led to a satisfactory level of understanding of this common psychopathology to this day despite having been proposed several decades ago,^12^ should not be considered the only possible explanation for the development of symptoms experienced by MDD patients. Indeed, aside from the aforementioned studies which support an involvement of the serotonergic system in MDD pathophysiology, there have been numerous publications reporting conflicting evidence (reviewed in References 13, 14, 15) as well as alternative hypotheses.^16^, ^17^, ^18^

Hence, focusing on the involvement of the serotonergic system in general, and SERT in particular, may therefore be seen as a particularly dogmatic approach to understanding MDD. However, strong evidence in the literature does indicate that this monoamine system is likely to be involved in the pathophysiology of depression, at least in a subpopulation of patients and further in‐depth analyses are therefore warranted.^13^, ^14^ This is especially true when considering the large number of possible interactions between different endogenous and exogenous factors and modulatory systems in the context of the pathogenesis of this highly prevalent and excruciating disorder.

For example, life stress has been repeatedly demonstrated as a highly relevant environmental variable determining vulnerability/resilience to the development of depression. Yet again, the molecular framework governing the intricate web of “nature and nurture” interactions remains incompletely explored.

Here, we identified the lipid raft‐associated molecule Flotillin‐1 (Flot1) as a novel protein interacting with SERT. Previously, Flot1 had been implicated in the internalization and membrane targeting of the dopamine transporter (DAT), which shares a high structural homology with SERT.^19^ As a result of this similarity, Flot1 was considered an obvious candidate protein for interacting with SERT, with the additional potential of this protein‐protein interaction being relevant in MDD.

Using a genetic mouse model for Flot1 deficiency and a combined molecular biological/biochemical, electrophysiological and behavioral approach we identified a role for Flot1 in the regulation of stress‐induced depression‐like behavior in the chronic corticosterone (CORT) paradigm. We provide evidence for a regulation of SERT expression and functional activity of serotonergic neurotransmission as an intermediate phenotype and delineate some of the molecular mechanisms involved.

## 2 METHODS AND MATERIALS

See Appendix S1, Supporting Information for a full description.

### 2.1 Animals

The generation of Flotillin‐1 knockout (Flot1 KO; background strain C57BL6/J) mice has been reported elsewhere.^20^ Serotonin transporter knockout (SERT KO) mice used for immunoprecipitation experiments were obtained by heterozygous crossings of the SERT‐Cre recombinase knock‐in mouse line.^21^ Adult KO and wild‐type (WT) littermate male and female mice (aged 10‐16 weeks) were used for all experiments. All animals were single‐housed in standard transparent laboratory cages under standard conditions.

### 2.2 Preparation of detergent‐resistant membrane fractions

Mice were sacrificed by neck dislocation and brains were rapidly dissected on ice. Membranes were subjected to density gradient centrifugation as described^22^ with minor modifications.

### 2.3 Protein isolation, nuclear fraction preparation and immunoprecipitation

Hippocampal tissue was powderized in liquid nitrogen and homogenized in a standard protein lysis buffer. Nuclear fractions were prepared from hippocampal tissue using the Nuclear Extraction Kit (Abcam, Cambridge, UK) by following the manufacturer's supplied protocol.

Immunoprecipitation was performed using an anti‐SERT antibody (SERT: 1:100, sc‐1458, Santa Cruz Biotechnology, Santa Cruz, California) according to an established protocol.^23^

### 2.4 Western blotting

A previously described procedure was used^24^ with the following primary antibody (SERT: 1:1000, sc‐1458, Santa Cruz Biotechnology; Flotillin‐1:1:1000, ab133497, Abcam; β‐actin: 1:2000, A0760‐40, US Biological, Salem, Massachusetts) which were incubated with membranes overnight at 4°C. Target protein densitometry values were normalized to those of the housekeeping protein to semi‐quantitatively determine protein levels.

### 2.5 Mass spectrometry (LC‐MS/MS)

After immunoprecipitation the eluate was subjected to electrophoretic separation of proteins as described earlier.^25^ Coomassie‐blue‐stained bands were excised from SDS‐PAGE gels digested with 10 ng/μL trypsin (Promega, Madison, Wisconsin) and subjected to LC‐MS/MS using an ion trap mass spectrometer (HCT ultra ETD II, Bruker Daltonics, Bremen, Germany) coupled with an Ultimate 3000 nano‐HPLC system (Dionex, Sunnyvale, California). MS spectra were recorded followed by 3 data‐dependent CID MS/MS spectra generated from 4 highest intensity precursor ions. The MS/MS spectra were interpreted with the Mascot search engine (version 2.4.0, Matrix Science, London, UK) against human the SwissProt database.

### 2.6 Fluorescence resonance energy transfer

For Fluorescence resonance energy transfer (FRET) analysis, YFP‐SERT (Y‐SERT) and CFP‐Flot1 (C‐Flot1) were co‐transfected in HEK293 cells using the calcium phosphate co‐precipitation method, as described elsewhere.^26^ A SERT construct tagged with CFP and YFP on its cytoplasmic N and C termini, respectively (to yield C‐SERT‐Y),^27^ was used as a positive control; CFP‐SERT and YFP‐myrpalm served as negative controls for FRET experiments.

The “three‐filter method” was implemented as previously described.^27^ Images were acquired using a 63× oil immersion objective under continuous usage of a gray filter (20% density). All experiments were conducted for individual transfections; 5 to 7 wide‐field images were captured during each experiment and 1 to 7 transfected cells per image included in the study. Distances *r* were calculated based on the Förster equation using the value of 4.92 nm as *R*_0_ for the CFP‐YFP FRET pair according to Patterson et al.^28^:

$$E=\frac{R_{0}^{\;6}}{\left(R_{0}^{\;6}+r^{6}\right)}$$

### 2.7 Quantitative real‐time PCR

mRNA extraction from manually dissected dorsal raphe nucleus (DRN) tissue was performed using the miRNeasy Mini Kit (Qiagen, Venlo, Netherlands) according to the manufacturer's protocol. Nine hundred nanogram of total RNA was used for cDNA synthesis with the DyNAmo cDNA Synthesis Kit (ThermoScientific, Waltham, Massachusetts). qRT‐PCR was performed using the SYBR Green MasterMix (Life Technologies, Carlsbad, California).

### 2.8 Behavioral tests

All behavior tests were carried out in order of increasing stressfulness^29^ and with at least 24 hours between individual tests (for a timeline see Figure S2). Behavioral tests were performed during the light phase of the light‐dark cycle, and mice were allowed to habituate to the experimental room for at least 30 minutes prior to testing.

#### 2.8.1 Sucrose preference test

The sucrose preference test (SPT) was adapted from Yu et al.^30^ (2007). Mice were first introduced to a 2% sucrose solution in tap water before the SPT in a training phase, by replacing the drinking water in their cages with aforementioned solution for 48 hours several days prior to testing. Following a return to tap water for 6 hours, mice were food‐ and water‐restricted for 18 hours before testing. During the 3 hours testing period, mice were offered the choice between sucrose and water. The sucrose preference (%) exhibited by each mouse, representing anhedonia‐like behavior, was calculated from the amount of sucrose consumed relative to total liquid consumption (mL).

#### 2.8.2 Forced swim test

The forces swim test (FST) was carried out according to a published procedure.^31^, ^32^, ^33^ Briefly, mice were placed in a beaker filled with water (22°C‐23°C) for 6 minutes and swimming behavior was filmed and automatically tracked employing the software Videotrack (Viewpoint, Champagne au Mont d'Or, France). Immobility, defined as the absence of any movement except for the ones required to keep the head above the water, was automatically recorded by the software using a movement threshold previously defined to match scoring by a trained experimenter. The percentage of time spent immobile as a measure of behavioral despair was evaluated during the final 4 minutes of the test.

#### 2.8.3 Light‐dark box test

The light‐dark box (LDB) test was adapted from previously published protocols.^34^ The test was conducted in a square arena (27.3 × 27.3 cm^2^) fitted with infrared beams for movement tracking as well as a “dark box” insert, featuring a small opening and covering half of the arena surface. Activity Monitor (MedAssociates Inc., St. Albans, Vermont) was used to track and analyze the movements of each mouse. The number of full‐body entries into the light compartment was used as parameter to indicate anxiety‐related behavior.

#### 2.8.4 Elevated plus maze

The elevated plus maze (EPM) apparatus consisted of a plus‐shaped maze elevated approximately 50 cm off the ground, with 2 open opposing arms and 2 closed opposing arms, the latter of which are surrounded by 20 cm high opaque walls. During a 5 minute trial, the number of full‐body entries of each mouse into the open arms was recorded by the software Videotrack (Viewpoint) and used as a parameter for the indication of anxiety‐related behavior.^35^, ^36^

#### 2.8.5 Open field test

In the open field test (OFT), total distance traveled during 1 hour was recorded using Activity Monitor (MedAssociates Inc.) in an arena (27.3 × 27.3 cm^2^) fitted with infrared beams to track the animals movements.

#### 2.8.6 Rotarod

Motor coordination was evaluated using an automated Rotarod system (MedAssociates Inc.)^37^ Here, mice were placed onto a rotating drum (speed: 4‐40 rpm, steadily increasing over 6 minutes) in 3 consecutive trials, and the latency to fall off was automatically recorded. The average latency to fall was calculated for each animal and employed as the relevant parameter for motor coordination in this test.

## 3 RESULTS

### 3.1 Flot1 and SERT are present in a complex and interact in vitro

**Figure 1:**
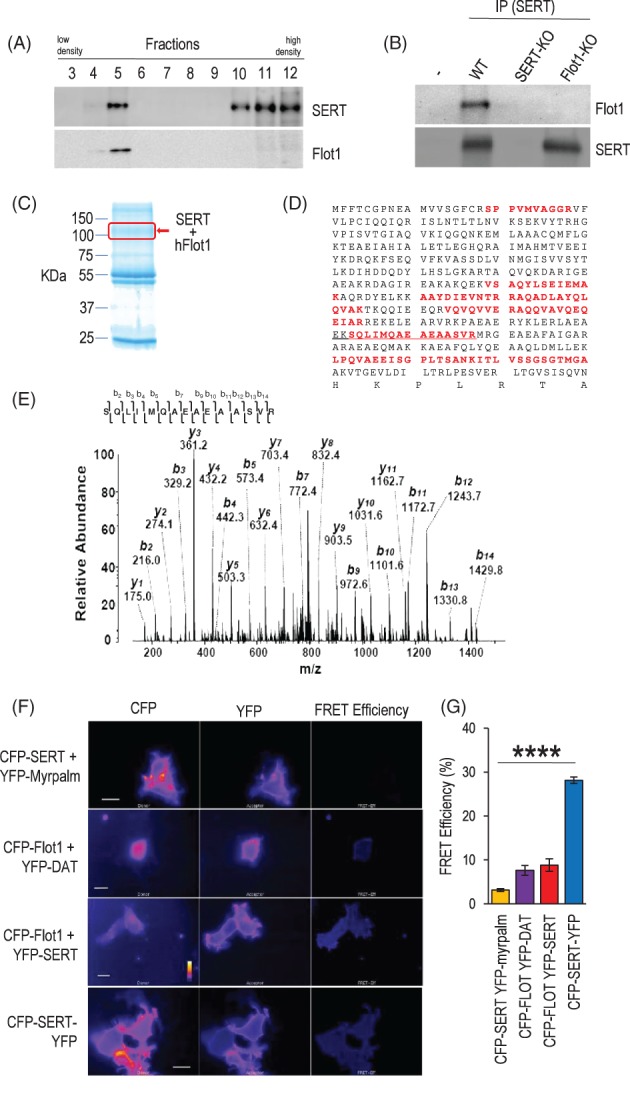
Flotillin‐1 (Flot1) and serotonin transporter (SERT) are present in a complex and directly interact in vitro. (A) Sucrose gradient centrifugation of mouse brain samples with subsequent western blotting reveals an overlapping pattern of enrichment of SERT and Flot1 among 12 fractions analyzed (fraction 1 and 2 are not shown as no signal was detected). (B) Co‐immunoprecipitation experiments of mouse brain lysate using SERT antibody (or no antibody as negative control) and subsequent western blot evaluation of the IP eluate determined immunoreactive bands corresponding to the expected molecular weights of SERT and Flot1. No bands were detected in tissue obtained from Flotillin‐1 knockout (Flot1 KO) and SERT‐KO mice. (C) Coomassie‐blue‐stained SDS‐PAGE gel analysis of SERT‐associated protein complex immunopurified using an anti‐SERT antibody with indication of bands with the expected molecular weight of Flot1 and SERT. (D) LC‐MS/MS indicated the presence of Flot1 in the IP eluate as demonstrated by the identified peptides (boldface type in red). (E) MS/MS spectrum obtained from doubly charged tryptic peptide at *m*/*z* 802.44 described the sequence SQLIMQAEAEAASVR (amino acids 303‐317, underscored) corresponding to human Flot1. (F) HEK‐293 cells were transfected with plasmids encoding CFP‐SERT and YFP‐myrpalm, CFP‐Flot1 and YFP‐SERT or YFP‐DAT and CFP‐SERT‐YFP and subjected to FRET analysis. The FRET efficiency was determined as described previously.^25^ All images are representative of 3 different experiments and corrected for background (scale bars: 10 μm). (G) Statistical analysis revealed a significant difference between groups (*F*_3,93_ = 107.0, *P* < .0001; *n* = 20‐30/group). Subsequent post hoc testing demonstrated a significant difference between the negative control (CFP‐SERT + YFP‐myrpalm) and CFP‐Flot1 + YFP‐SERT (*P* = .0012). Data are depicted as mean ±SEM; significant effects are depicted as: ^****^ *P* < .0001

Considering the prominent membrane localization of SERT, we carried out a sucrose gradient centrifugation of mouse brain membranes to identify a possible interaction with Flot1. A striking co‐localization of SERT and Flot1 in fraction 5 was observed (Figure 1A), indicating that in the mouse brain SERT associates with Flot1‐positive membrane fractions as previously described for heterologously expressed SERT.^38^ We therefore sought to further elucidate the nature of physical contact between Flot1 and SERT using immunoprecipitation (IP) experiments. When using a SERT antibody to immunoprecipitate potentially interacting protein partners from mouse brain lysate, a band corresponding to the immunochemical identity of Flot1 was readily detected by subsequent western blot analysis (Figure 1B). We next employed a mass spectrometrical approach to additionally confirm the Co‐IP result. To this end, SERT complexes were purified from HEK293T cells expressing GFP‐SERT by using an anti‐GFP antibody and the obtained pull‐down fraction was separated by SDS‐PAGE.^25^ The Coomassie blue‐stained bands were excised (Figure 1C) and in‐gel tryptic digestion for MS analysis was performed. LC‐MS/MS unambiguously identified human Flot1 (Uniprot ID, O75955; http://www.uniprot.org/uniprot/O75955) from SERT complexes with 9 specific peptides (Figure 1D). A representative MS/MS spectrum with fragmented b‐ and y‐ions described the sequence SQLIMQAEAEAASVR which was assigned to human Flot1 by Mascot database (http://www.matrixscience.com/) searches (Figure 1E). These data rendered additional support to the results obtained by sucrose gradient and co‐IP analysis, suggesting that Flot1 and SERT can be localized in the same protein complex in the brain.

We next resorted to FRET analysis to address the question whether Flot1 might be directly interacting with SERT. In an in vitro approach, CFP‐ and YFP‐labeled proteins were co‐expressed in HEK293T cells. A SERT fusion protein with both CFP and YFP, each individually attached to one intracellular terminus (CFP‐SERT‐YFP),^27^ yielded the expected strong resonance energy transfer (FRET efficiency 28.15 ± 0.73) and served as positive control (Figure 1F). CFP‐SERT and YFP‐myrpalm served as negative control and yielded a low resonance energy transfer (FRET efficiency 3.14 ± 0.34, Figure 1F). FRET experiments employing CFP‐Flot1 and YFP‐SERT provided evidence that the 2 proteins were in a physical vicinity which allowed for resonance energy transfer (Figure 1F), which was in magnitude significantly different from that of the negative control (Figure 1G). Interestingly, the examination of CFP‐Flot1 and YFP‐DAT yielded a comparable mean FRET efficiency (YFP‐SERT: 8.85 ± 1.41; YFP‐DAT: 7.62 ± 1.11; Figure 1G). This finding is noteworthy considering the high degree of structural similarity between SERT and DAT^39^ and the fact that DAT and Flot1 have been previously reported to be physical interaction partners.^19^ The present FRET results are in line with the immunochemical and mass spectrometrical analyses described above and jointly suggest an interaction between SERT and Flot1. While the herein reported observations propose this interaction to be mediated on the basis of a direct, physical contact between the 2 proteins, the possibility of additional, intermediary proteins being present within the protein complex cannot be rejected.

### 3.2 Flot1 KO do not present with abnormal emotional behavior

**Figure 2:**
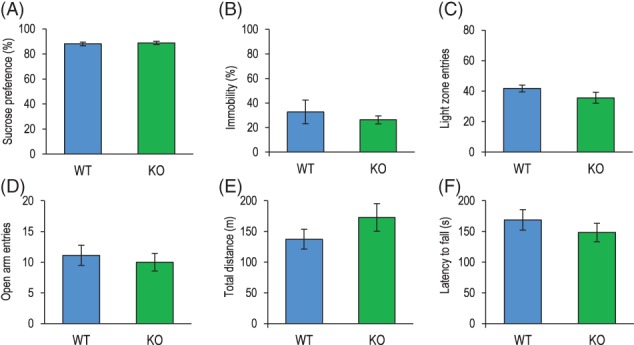
No overt behavioral deficits are detectable in Flotillin‐1 knockout (Flot1 KO) mice under baseline conditions. Unaltered depression‐like behavior in (A) the sucrose preference test (SPT), as reflected in the percentage of sucrose preference relative to total liquid consumption (*t* test: *t*_16_ = 0.43, *P* = .68; *n* = 8‐10/group) and (B) the forced swim test (FST), represented by percentage immobility in the last 4 minutes of the test (Welch's *t* test: *t*_8.68_ = 0.63, *P* = .54; *n* = 8‐9/group). Number of entries into (C) the light zone of the LD box (*t* test: t_16_ = 1.36, *P* = .19; *n* = 8‐9/group) and (D) the open arms of the elevated plus maze do not reveal differences between genotypes (*t* test: *t*_14_ = 0.06; *P* = .96; *n* = 8/group). (E) Locomotor activity in the OF (*t* test: *t*_16_ = 1.22, *P* = .24; *n* = 8‐10/group) and (F) motor coordination in the RR are comparable between genotypes (*t* test: *t*_15_ = 0.47, *P* = .65; *n* = 8‐9/group). Data are depicted as mean ± SEM

Considering the obtained evidence for a co‐localization of Flot1 and SERT in the same protein complex, we set out to explore whether absence of Flot1 modulated SERT expression and/or affective behaviors. To this end we used Flot1 KO mice as model of genetic Flot1 deficiency and characterized depression‐like behavior within a well‐defined battery of tests for behavioral phenotyping. No differences in anhedonic behavior in the Sucrose Preference Test or behavioral despair in the FST between adult Flot1 KO and wild‐type littermate (WT) controls were observed (Figure 2A,B). Furthermore, anxiety‐like behavior was indistinguishable between Flot1 KO and WT mice in the light‐dark box and the elevated plus maze (Figure 2C,D). Assessment of general exploratory and locomotor behavior (open field) as well as motor coordination (Rotarod) did not provide evidence for differences between genotypes (Figure 2E,F), indicating no overt behavioral abnormalities in Flot1 KO.

### 3.3 SERT expression and activity of serotonergic neurons is unaltered in Flot1 KO mice

**Figure 3:**
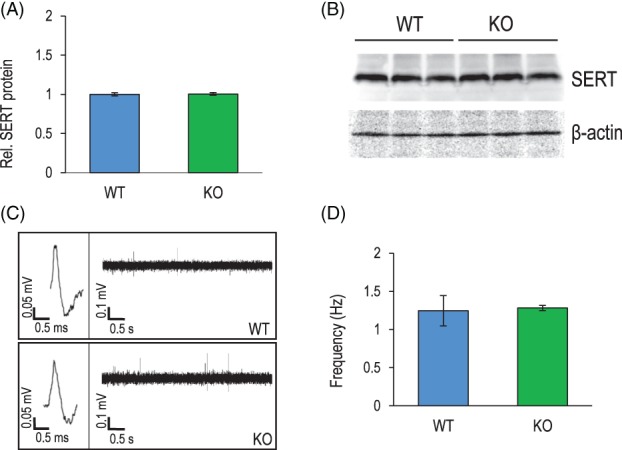
Flotillin‐1 knockout (Flot1 KO) mice present with unaltered serotonin transporter (SERT) expression, function and activity of serotonergic neurons under baseline conditions. (A) Comparable hippocampal SERT protein levels in Flot1 KO and wild‐type (WT) mice as quantified from the respective western blot bands (B) (*t* test: *t*_4_ = 0.42, *P* = .70; *n* = 3/group). (C and D) in vivo recordings of extracellular spikes in dorsal raphe nucleus (DRN) of Flot1 KO and WT mice do not indicate group differences in firing rates (*t* test: *t*_10_ = 0.0, *P* = .99; *n* = 6/group). Data are depicted as mean ±SEM

We next set out to examine the consequences of Flot1 deficiency of expression of SERT. The evaluation of SERT levels by western blot revealed comparable amounts of hippocampal SERT protein in Flot1 KO and WT mice (Figure 3A,B). In order to further characterize SERT function in our model in vivo, we employed an electrophysiological approach based upon the investigation of the spontaneous activity of midbrain dorsal raphe serotonergic neurons. Extracellular recordings of spikes with the characteristics of serotonergic neurons^40^ revealed no differences between genotypes for the mean firing frequency (Figure 3C,D), confirming that the functional state of serotonergic neurotransmission in the intact animal was not impaired in Flot1 KO.

### 3.4 Genetic Flot1 deficiency renders mice more susceptible to the depressogenic effects of chronic CORT and differentially regulates the response of the serotonergic system

**Figure 4:**
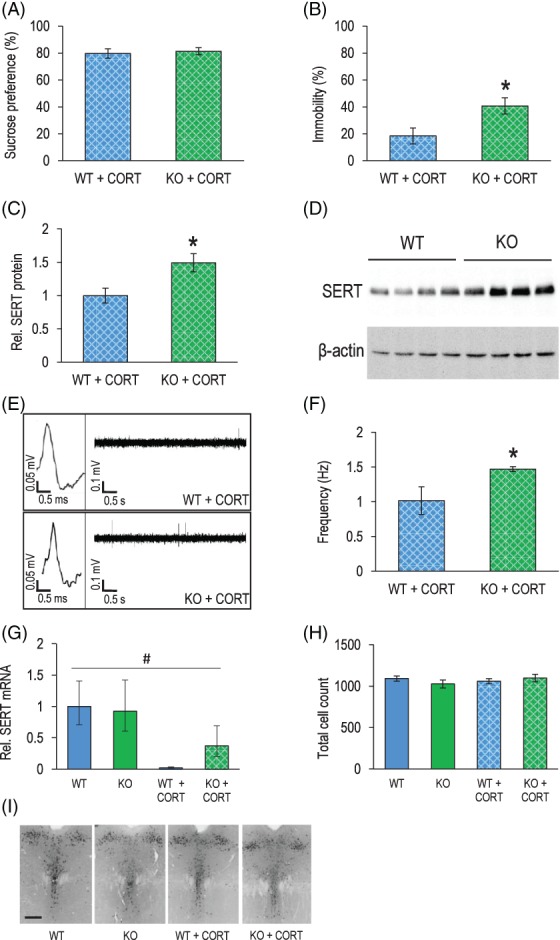
Depression‐like behavior and serotonergic neurotransmission are differentially impacted by long‐term chronic corticosterone (CORT) treatment in Flotillin‐1 knockout (Flot1 KO) mice. (A) No significant differences were observed in anhedonic behavior, as measured by percentage sucrose preference in the sucrose preference test (SPT), between Flot 1 KO and wild‐type (WT) after chronic CORT treatment (*t* test: *t*_16_ = 0.40, *P* = .70; *n* = 8‐10/group). (B) Behavioral despair (represented by percentage immobility) during the FST was significantly higher in Flot1 KO mice compared to controls after CORT (*t* test: *t*_15_ = 2.54; *P* = .02; *n* = 7‐10/group). (C) Augmented hippocampal serotonin transporter (SERT) protein levels after chronic CORT treatment in Flot1 KO as compared to WT mice revealed by quantification of (D) respective Western Blot bands (*t* test: *t*_6_ = 2.78, *P* = .03; *n* = 4/group). (E) More extracellular spikes in the dorsal raphe nucleus (DRN) of chronically CORT‐treated Flot1 KO than in WT mice resulting in (F) a significantly higher firing rate during in vivo recordings (*t* test: *t*_10_ = 2.18, *P* = .05; *n* = 6/group). (G) Two‐way ANOVA demonstrated a significant treatment × genotype interaction on SERT mRNA levels in the DRN of Flot1 KO and WT mice under baseline conditions and after chronic CORT treatment (genotype × treatment: *F*_1,22_ = 7.31, *P* = .01; *n* = 6‐7/group). Post hoc Tukey's test revealed that after CORT treatment there was a significant difference between genotypes (*P* = .006) but not at baseline (*P* = .99), while also showing that CORT treatment significantly impacted SERT mRNA levels in WT (*P* = .0002) but not in KO (*P* = .47). (H and I) Immunohistochemical analysis of the dorsal raphe nuclei revealed no differences in total number of 5‐HT‐positive cells between Flot1 KO and WT in both treatment groups (genotype: *F*_1,14_ = 0.11, *P* = .75; treatment: *F*_1,14_ = 0.24, *P* = .63; genotype × treatment: *F*_1,14_ = 1.63, *P* = .22; *n* = 4‐5/group). Representative images are shown (scale bar: 100 μm). Data are depicted as mean ±SEM; main effect of genotype depicted as: ^*^ *P* < .05; interaction genotype × treatment depicted as: ^#^ *P* < .05

Considering the proposed role of SERT as the genetic backbone determining the impact of environmental factors on the development of depression, we resorted to the chronic CORT paradigm as an experimental animal model for long‐term stress exposure in a separate cohort of Flot1 KO and wildtype littermates. While differences in sucrose preference were not observed between Flot1 KO and control mice after CORT exposure (Figure 4A), in contrast Flot1 KO mice responded with a higher display of depression‐like behavior in the FST than WT after chronic CORT exposure (Figure 4B).

No differences between groups were observed after CORT treatment in the OF, LD, EPM and RR tests (data not shown). Interestingly, hippocampal SERT protein expression was also higher in CORT‐treated Flot1 KO than WT mice (Figure 4C,D). Correspondingly, genotype‐dependent recordings of the serotonergic firing activity in the DRN of CORT‐treated mice were obtained (Figure 4E,F). These results, attesting to a distinct impact of chronic CORT treatment on behavior and the functional state of the serotonergic system in Flot1 KO as compared to WT mice, were also paralleled at the SERT mRNA level. Here, a striking reduction in SERT mRNA was observed upon CORT treatment in both genotypes; however, with a significantly blunted response seen in Flot1 KO mice compared to WT (Figure 4G). An immunohistochemical approach was selected in order to experimentally address the possibility that this finding merely reflected a reduction in the number of serotonergic neurons. No significant effects of treatment or genotype on the number of serotonergic neurons in Flot1 KO and WT before and after CORT treatment were observed (Figure 4H,I). Overall, these results suggest a differential response of Flot1 KO in the behavioral and serotonergic responses to long‐term chronic CORT exposure.

### 3.5 The expression of molecular constituents of the glucocorticoid receptor nuclear translocation machinery changes distinctively in response to chronic CORT treatment in Flot1 KO and WT mice

**Figure 5:**
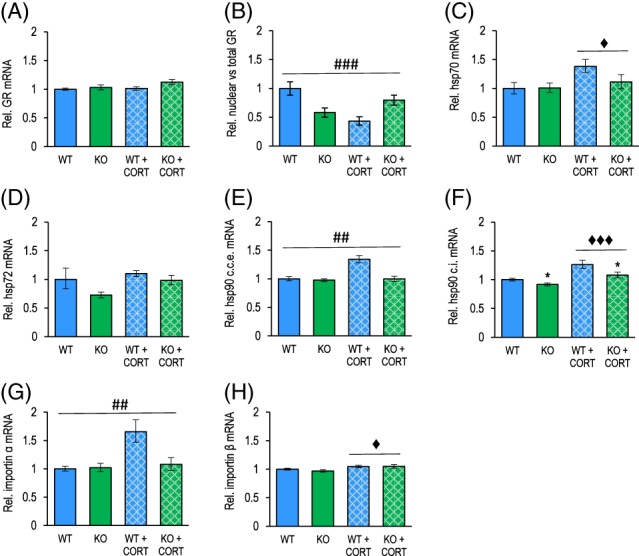
Distinct profile of glucocorticoid receptor nuclear translocation and expression of molecular constituents of the nuclear translocation machinery in response to chronic corticosterone treatment in Flot1 KO. (A) Chronic CORT treatment results in comparable GR mRNA levels in the DRN of Flot1 KO and WT mice (genotype: *F*_1,23_ = 3.80, *P* = .06; treatment: *F*_1,23_ = 2.12, *P* = .16; genotype × treatment: *F*_1,23_ = 1.21, *P* = .28; *n* = 6‐8/group). (B) Relative distribution of GR protein in the cytosolic fraction vs total GR protein in hippocampal tissue of Flot1 KO and WT mice under control conditions and after chronic CORT treatment (genotype × treatment: *F*_1,18_ = 17.57, *P* = .001; *n* = 4‐6/group). Post hoc analysis showed that relative cytosolic GR differed between genotypes both at baseline (*P* = .003) and after chronic CORT (*P* = .02), but that chronic CORT had a strong impact in WT (*P* = .0003) while not significantly affecting KO (*P* = .14). Two‐way ANOVA reports for DRN mRNA levels of (C) Hsp70 (genotype: *F*_1,21_ = 1.23, *P* = .28; treatment: *F*_1,21_ = 5.07, *P* = .04; genotype × treatment: *F*_1,21_ = 1.40, *P* = .25; *n* = 6‐7/group), (D) Hsp72 (genotype: *F*_1,23_ = 3.63, *P* = .07; treatment: *F*_1,23_ = 3.09, *P* = .09; genotype × treatment: *F*_1,23_ = 0.86, *P* = .36; *n* = 5‐8/group), (E) Hsp90 (c.c.e.: constitutively expressed form; genotype × treatment: *F*_1,21_ = 11.38, *P* = .003; post hoc analysis: WT baseline vs WT post CORT: *P* = .0001, KO baseline vs KO post CORT: *P* = .98; baseline WT vs baseline KO: *P* = .98, post CORT WT vs post CORT KO: *P* = .0002; *n* = 5‐8/group) (F) Hsp90 (c.i.: inducible form; genotype: *F*_1,23_ = 7.60, *P* = .01; treatment: *F*_1,23_ = 20.12, *P* = .0002; genotype × treatment: *F*_1,23_ = 0.65, *P* = .43; *n* = 5‐8/group), (G) Importin‐α (genotype × treatment: *F*_1,21_ = 5.81, *P* = .03; post hoc analysis: WT baseline vs WT post CORT: *P* = .003, KO baseline vs KO post CORT: *P* = .98; baseline WT vs baseline KO: *P* = .99, post CORT WT vs post CORT KO: *P* = .02; *n* = 5‐7/group), (H) Importin‐β (genotype: *F*_1,23_ = 0.30, *P* = .59; treatment: *F*_1,23_ = 5.81, *P* = .02; genotype × treatment: *F*_1,23_ = 0.47, *P* = .50; *n* = 5‐8/group). Data are depicted as mean ±SEM; main effect of treatment depicted as: ^♦^ *P* < .05; ^♦♦♦^ *P* < .001; interaction genotype × treatment depicted as: ^##^ *P* < .01; ^###^ *P* < .001

The observed augmented behavioral response of Flot1 KO mice to long‐term CORT treatment led us to hypothesize that this enhanced sensitivity might relate to altered expression of glucocorticoid receptors (GR) in Flot1 KO mice. qRT‐PCR analysis revealed no effect of genotype on mRNA levels of GR (Figure 5A). We next considered the possibility that, rather than total gene expression, protein levels and/ or nuclear translocation of GR in response to chronic CORT could show genotype‐dependency. We therefore determined the patterns of subcellular localization of GR together with total protein levels in Flot1 KO and WT mice. Fractionation analysis and subsequent western blotting revealed that chronic CORT treatment reduced total GR protein levels and additionally induced a subcellular re‐distribution of GR in a genotype‐dependent manner (Figures 5B and S1A‐C): The significant shift in relative nuclear GR levels seen in WT because of chronic CORT appear to be blunted in Flot1 KO mice.

Aiming to examine the nature of this differential regulation of nuclear transport in Flot1 KO as compared to WT mice upon chronic CORT exposure we set out to examine the expression of molecular elements of the GR translocation machinery in Flot1 KO and WT mice. We focused on the 2 main chaperone systems known to contribute to the transport and activity of GR—Hsp70 and Hsp90 (reviewed in Reference 41)—and the nuclear import receptors importin α and importin β, central to the nuclear translocation of GR.^42^, ^43^ For Hsp70 mRNA levels (Figure 5C), 2‐way ANOVA revealed a significant effect of treatment, resulting in increased expression after CORT in both groups. For its major inducible counterpart Hsp72, no significant effects of genotype or treatment were observed (Figure 5D), although a trend for reduced expression in Flot1 KO mice in both conditions was noted. Relative levels of the constitutively expressed (c.c.e.) Hsp90 showed a significant treatment × genotype interaction, with differences between genotypes emerging only after CORT treatment because of CORT appearing to be ineffective in Flot1 KO. For the inducible cytosolic (c.i.) form of Hsp90, significant effects of genotype and treatment were observed (Figure 5E,F). A significant interaction of treatment × genotype factors was observed for levels of importin α, which again appears to confirm that CORT treatment does not affect Flot1 KO in the same way as WT mice, and with differences between the genotypes emerging after the chronic CORT treatment for this reason. Finally, only a modest treatment effect for importin β was found (Figure 5G,H). Overall these results pointed toward a Flot1‐dependency in the nuclear transport of GR upon long‐term CORT exposure.

## 4 DISCUSSION

Since the discovery of SSRIs more than 4 decades ago, the serotonin transporter has undoubtedly been the “poster child” of pharmaceutical companies developing and commercializing antidepressant drugs. However, only about 40% to 60% of depressed patients exhibit a treatment response after first‐line treatment with these drugs with remission rates ranging from 30% to 45%.^44^

An increasing body of evidence demonstrates the role of environmental influences as susceptibility factors contributing to the development of depression and the large variability in the responsiveness to antidepressant medications. However, little is known about the nature of the convergence of these exogenous factors on the availability of SERT and the endogenous disposition of its regulation.

Here we identified Flotillin‐1 as novel physical interaction partner of SERT in the mouse brain and demonstrate a role for Flot1 as a mitigating factor in chronic CORT‐induced SERT regulation and depression‐like behavior. The striking co‐localization and physical interaction of SERT and Flotillin‐1 which we demonstrate using biochemical, immunochemical and mass spectrometrical assays extends previous reports describing that Flot1 and SERT both reside in SCAMP2‐containing structures.^45^ Further, our findings are in line with reports on the dopamine transporter (DAT), a neurotransmitter transporter with a high degree of structural similarity to SERT, which has been demonstrated to co‐localize with Flotillin‐1 in the same protein complex.^19^ A comprehensive behavioral characterization of Flot1 KO mice revealed no overt phenotypic distinctions from WT littermates under baseline conditions, indicating that the genetic disruption by itself is not sufficient to induce behavioral abnormalities and/or that efficient compensatory mechanisms might be at work. The concomitant demonstration of unaltered SERT expression and activity of DRN neurons in vivo further indicates that, despite the physical interaction between the 2 molecules, the mere genetic depletion of Flot1 did not lead to an apparent dysbalance in the serotonergic neurotransmission in vivo.

Based upon these observations, we considered that Flot1 might be relevant within the “gene × environment” interaction framework which is largely considered to account for the multifactorial origins of psychiatric disorders, including depression. We therefore tested whether Flot1 deletion may constitute a vulnerability factor for behavioral and serotonergic abnormalities upon exposure to adverse environmental conditions.^46^, ^47^ A number of environmental adversities are known to impact on the given genetic makeup of an individual and contribute to the liability for depression. Stress exposure is recognized as the major external contributing factor to risk for the development of psychopathologies, with an enhanced sensitivity exhibited during early life but highly relevant throughout adulthood.^48^, ^49^, ^50^ In order to evaluate how environmental challenges impinge on the genetic background of Flot1 deficiency to lead to depression‐like behavior, we subjected Flot1 KO mice and WT littermates to chronic CORT treatment. We found that chronic CORT treatment resulted in augmented immobility in the FST in Flot1 KO compared to WT mice but did not affect the SPT. This finding is noteworthy as it suggests a degree of specificity in the sensitivity of Flot1 KO mice in the behavioral repertoires typically associated with a depression‐like phenotype. While the SPT is considered to reflect anhedonic‐like behavior, the performance in the FST is generally considered to indicate behavioral despair. Depression in human patients presents as a heterogeneous disorder in humans with a display of a spectrum of symptoms in varying combinations which is likely to represent the different subtypes of the disorders. In light of the restricted profile of tests available for the detection of the respective phenotypes in preclinical models of the disease it is expectable that also in experimental animals behavioral alterations would be detectable in some, but not other paradigms. This consideration also extends to the analysis of anxiety‐like behavior in the LD and EPM, as in some, but not all depressed patients, pathological anxiety constitutes a major clinical symptom.

The fact that CORT treatment had no effect on locomotor activity and motor coordination (OFT and RR) on the other hand, is most relevant in terms of a performance control for the FST as it suggests that the observed effect indeed reflects an increase in despair‐like behavior rather than a general impact on motor activity after CORT.

Interestingly, this specific susceptibility for the development of despair‐like behavior in Flot1 KO mice under challenge was paralleled by a concomitant alteration in SERT expression and in the activity of serotonergic neurons which could lead to the speculation that altered SERT levels and function may be a distinct feature also of a subgroup of human patients suffering to depression, a hypothesis which remains to be tested in future translational and clinical studies.

Most striking was the dramatic reduction of SERT mRNA levels upon chronic CORT exposure with a significantly diminished response seen in Flot1 KO. We excluded the possibility that the detected changes in SERT levels merely reflected an alteration in the number of serotonergic neurons by independent evaluation of 5‐HT‐positive cells in the DRN, which showed no influence by either genotype or treatment. These substantial differences in SERT expression between genotypes upon chronic CORT administration, as well as activity of serotonergic neurons in KO mice suggested a critical interaction of Flot1 with the endogenous stress response system. Indeed, Flot1 has been described to co‐localize with a discrete pool of GR at the plasma membrane where the two proteins were found to be present in a complex.^51^ The low‐affinity GR is known to transduce the effects of heightened CORT levels by the translocation of the inactive, cytosolic form of the receptor to the nucleus upon binding of CORT and subsequent rearrangement of the accompanying chaperone machinery. Furthermore, genetic variants of GR have been associated with alterations in the stress response and the risk for development of psychiatric disorders.^52^, ^53^, ^54^ Here, we report that the subcellular distribution of the protein changed in response to chronic CORT, with differential enrichment in the nuclear vs the cytosolic fractions in a Flot1‐dependent manner. While acute exposure to corticosteroids is known to result in an enhancement of nuclear GR, a cytosolic accumulation of GR has been found to relate to enhanced corticosterone levels and associated depressogenic effects of chronic social stress experience.^55^ Accordingly, while in WT mice CORT treatment reduced nuclear GR in relation to the cytosolic protein, the reverse effect was found in Flot1 KO mice which showed more cytosolic GR upon long‐term CORT exposure. When we next examined the levels of several molecular elements of the nuclear translocation machinery, a pattern of overall up‐regulation in WT mice after CORT treatment emerged, which appears to be blunted in Flot1 KO. These results suggested a potential disruption of nuclear translocation of GR protein in response to long‐term CORT exposure in Flot‐1 KO mice and led us to hypothesize a relevance for this. In conclusion we here establish Flot1 as novel SERT‐interacting protein and comprehensively characterize the serotonergic and behavioral phenotype of Flot1 KO mice. We further suggest that the genetic depletion of Flot1 augments the sensitivity for the development of chronic CORT‐induced behavioral despair and alters the concomitant regulation of SERT transcription. While the genotype‐dependent effects of chronic CORT treatment may not be a direct consequence of the demonstrated Flot1‐SERT interaction at the plasma membrane, they do support a significant role of Flot1 in the regulation of long‐term CORT‐induced behavioral, functional and molecular alterations of the serotonergic system. Future studies will be required to elucidate the role of Flot1 on SERT activity at the plasma membrane, where a possible involvement of Flot1 in SERT endocytosis is suggested by related observations in the dopamine and norepinephrine transporters.^19^, ^56^

## Supporting information


**Appendix S1.** Methods and materials

**Figure S1.** Analysis of hippocampal glucocorticoid receptor protein levels by subcellular compartment in response to chronic corticosterone in Flot1 KO and WT mice. Chronic CORT treatment results in a significant reduction in relative GR protein in the (A) cytosol (genotype: *F*_1,20_ = 2.020, *P* = .171; treatment: *F*_1,20_ = 9.805, *P* = .005; genotype × treatment: *F*_1,20_ = 1.043, *P* = .319; n = 6/group) and (B) the nucleus (genotype: *F*_1,21_ = 1.041, *P* = .319; treatment: *F*_1,21_ = 5.699, *P* = .026; genotype × treatment: *F*_1,21_ = 4.024, *P* = .058; *n* = 4‐8/group) in both Flot1 KO and WT mice, which was also reflected in (C) total relative GR levels in the hippocampus (genotype: *F*_1,18_ = 3.103, *P* = .095; treatment: *F*_1,18_ = 7.778, *P* = .0121; genotype × treatment: *F*_1,18_ = 3.652, *P* = .0721; *n* = 4‐6/group). Data are depicted as mean ±SEM; main effect of treatment depicted as: ^♦^ *P* < .05; ^♦♦^ *P* < .01

**Figure S2.** Timeline displaying the sequence of behavioral testing. A 24 hour time interval was observed between the individual behavioral tests which were carried out in order or increasing stressfulness
