# Supplementary material for: Flotillin‐1 interacts with the serotonin transporter and modulates chronic corticosterone response
Source: Genes Brain Behav. 2018 May 20;18(2):e12482. doi: 10.1111/gbb.12482 (PMC6392109; doi:10.1111/gbb.12482)
Supplement: Supplementary file 1 — Appendix S1. Methods and materials [file GBB-18-na-s002.docx]

**Methods and Materials**

**Animals**

The generation of Flotillin-1 knockout (Flot1 KO) mice has been reported elsewhere (Ludwig *et al.* 2010). Heterozygous breeding pairs were provided by the laboratory of Benjamin J. Nichols and used for the establishment of a Flot1 KO mouse colony at the breeding facility of the Medical University of Vienna (Himberg, Austria). Serotonin transporter knockout (SERT KO) mice used for immunoprecipitation experiments were obtained by heterozygous crossings of the SERT-Cre recombinase knock-in mouse line (Zhuang *et al.* 2005).

All animals were housed in standard transparent laboratory cages in a temperature-controlled colony room (22 ± 1°C) and provided with standard laboratory food and water *ad libitum* unless noted otherwise. Mice were maintained on a 12-hour light/dark cycle (with lights on at 6:00 am, 200-220 lux inside the cages). Adult male and female mice (10-12 weeks old at onset of experiments) were used for all experiments. Separate cohorts of mice were used for behavior experiments and molecular experiments so as to exclude the potential for behavior testing itself having an impact on any of the examined physiological parameters in molecular experiments. After a pilot experiment revealed no significant *genotype x sex* interaction in any behavior tests, all behavior experiments were carried out in males while females were used for sample collection for molecular experiments. The same cohort of male animals was tested under baseline conditions and after CORT treatment.

Animal experiments described in this study were approved by the national ethical committee on animal care and use (Bundesministerium für Wissenschaft und Forschung; proposal no. GZ-66.009/0078-WF/V/3b/2016) and carried out in line with the Austrian Act on Animal Experiments 2012 (Tierversuchsgesetz 2012).

##### **Preparation of detergent-resistant membrane fractions**

Mice were sacrificed by neck dislocation and brains were rapidly dissected on ice. Membranes were subjected to density gradient centrifugation as described (Schuck *et al.* 2003) with the following modifications: After density gradient centrifugation, membranes were resuspended in 0.5 mL of buffer (50 mM Tris, pH 7.4, 2 mM EDTA, 150 mM NaCl), and homogenized by 10 aspiration cycles each through a 20- and a 27-gauge needle. Buffer (0.5 mL) containing 0.5% Triton X-100 was added. Extraction was done on ice for 30 min. The lysate (0.7 mL) was adjusted to 42% (wt/wt) sucrose by adding 1.4 mL of 59% sucrose in buffer, overlaid with 6 mL of 35% and 4 mL of 5% sucrose, and centrifuged at 217,000 G  for 18 h. Samples were fractionated in 1-mL aliquots from the top (fraction 1) to the bottom.

**Protein isolation, nuclear fraction preparation and immunoprecipitation**

Brain tissue was powderized in liquid nitrogen and homogenized in a protein lysis buffer containing 10 mM Tris-HCl, pH 7.5, 150 mM NaCl, 1% SDS, 0.5% Triton X100, 1 mM EDTA, 10 mM NaF, 5 mM Na_4_O_2_P_7_, 10 mM Na_3_VO_4_ and protease inhibitor cocktail (1×, Roche Diagnostics, Mannheim, Germany). After sonication for 5 cycles × 5s, the suspension was left at 4°C on a rotator for 30 minutes and centrifuged at 14,000 G for 30 min at 4°C. The supernatant was immediately transferred and was quantified using Pierce BCA assay Kit (ThermoScientific, Waltham, MA, USA). The standard curve was generated using kit-provided Bovine Serum Albumin with a concentration of 2mg/mL. The samples were analyzed in triplicates (microplate procedure: 25 µL sample + 200 µL BCA working reagent and incubated at 37°C for 30 minutes) and the concentration was determined by absorbance reading at 595 nm using a Synergy H4 Hybrid Reader spectrophotometer (Szabo-Scandic HandelsgmbH & Co KG, Vienna, Austria). Nuclear Fractions were prepared from hippocampal tissue using the Nuclear Extraction Kit (Abcam, Cambridge, UK) by following the manufacturer’s supplied protocol.

Immunoprecipitation was performed by a standard procedure using an anti-SERT antibody (SERT: 1:100, sc-1458, Santa Cruz Biotechnology, Santa Cruz, California) which was incubated with protein lysate at 4°C overnight, coupled with 30 µL Protein A/G agarose beads for 2 hours at 4°C, then washed 5 times with 1×PBS with 1% Triton X-100 before elution.

**Western Blotting**

5μL loading buffer (ThermoScientific, Waltham, MA, USA) was added to 25 μg of total protein for use in SDS-PAGE electrophoresis (10 % SDS mini-gel and 5% stacking gel; electrophoresis settings: 80 V, 1 h 45 min using a Mini-Protean System, Bio-Rad Laboratories, Hercules, CA, USA). Proteins were transferred to PVDF membranes (Millipore, Billerica, MA, USA) by running at 250 mA for 1 h 30 min. Membranes were blocked by incubating with 5% nonfat dry milk in 100 mM Tris, pH 7.5, 150 mM NaCl, and 0.1% Tween 20 (TTBS) for 1 h. Membranes were then incubated with diluted primary antibody (SERT: 1:1000, sc-1458, Santa Cruz Biotechnology, Santa Cruz, California; Flotillin-1: 1:1000, ab133497, Abcam, Cambridge, UK; β-actin: 1:2000, A0760-40, US Biological, Salem, MA, USA) overnight at 4°C, rinsed three times with TTBS, and incubated for 1 h at room temperature with horseradish peroxidase-conjugated secondary antibody. Immunoreactivity was visualized by enhanced chemiluminescence Pierce ECL substrate (ThermoScientific, Waltham, MA, USA). Detectable molecular masses were determined by running standard protein markers (ThermoScientific, Waltham, MA, USA) ranging from 10 to 250 kDa. Chemiluminescent imaging with a FluorChem HD2 and accompanying software (Alpha Innotec, Kasendorf, Germany) were used for quantification: Target protein densitometry values were normalized to housekeeping genes to semi-quantitatively determine protein levels, as previously described (Griesauer *et al.* 2014).

**Mass spectrometry (LC-MS/MS)**

After immunoprecipitation the eluate was subjected to electrophoretic separation of proteins. Coomassie-blue-stained bands were excised from SDS-PAGE gels, destained with 50% acetonitrile in 50 mM ammonium bicarbonate, and dried in a speed-vacuum concentrator. After reduction and alkylation of Cys, gel pieces were washed and dehydrated. Dried gel pieces were swollen with 25 mM ammonium bicarbonate (pH 8.0) containing 10 ng/µL trypsin (Promega, Madison, WI, USA) and incubated at 37°C for 18 h. Digested peptides were extracted with 50% acetonitrile in 5% formic acid and concentrated in a speed vacuum concentrator for LC-MS/MS.

An ion trap mass spectrometer (HCT ultra ETD II, Buker Daltonics, Bremen, Germany) coupled with an Ultimate 3000 nano-HPLC system (Dionex, Sunnyvale, CA, USA) was used for LC-MS/MS data acquisition. A PepMap100 C-18 trap column (300 μm × 5 mm) and PepMap100 C-18 analytic column (75 μm × 150 mm) were used for reverse phase (RP) chromatographic separation with a flow rate of 300 nL/min. The two buffers used for the RP chromatography were 0.1% formic acid (FA) in water (buffer A) and 0.08% FA in acetonitrile (buffer B) with a 125 min gradient (4-30% B for 105 min, 80% B for 5 min and 4% B for 15 min). Eluted peptides were then directly sprayed into the mass spectrometer to record peptide spectra over a mass range of m/z 350-3500 and MS/MS spectra in information-dependent data acquisition over the mass range of m/z 100-2800. Repeatedly, MS spectra were recorded followed by three data-dependent CID MS/MS spectra generated from four highest intensity precursor ions. The MS/MS spectra were interpreted with the Mascot search engine (version 2.4.0, Matrix Science, London, UK) against human Swissprot database. Database searches were performed with a mass tolerance of 50 ppm and an MS/MS tolerance of 0.5 Da, and 3 missing cleavage sites, carbamidomethylation of cysteine and oxidation of methionine were allowed.

##### **DNA constructs, cell culture and transfection**

HEK293 cells were grown in Dulbecco's Modified Eagle’s Medium (DMEM), supplemented with 10% fetal calf serum, 1% penicillin/streptomycin and maintained at 37°C, in a 5% CO2 humidified atmosphere, on standard plastic culture ware. A Flot1 cDNA construct tagged with GFP (laboratory of [Benjamin J. Nichols](http://jcb.rupress.org/search?author1=Benjamin+J.+Nichols&sortspec=date&submit=Submit), MRC, Cambridge; (Glebov *et al.* 2006)) was cloned into the CFP/YFP-N1 vector (Clontech, Mountain View, CA, USA) without the GFP sequence. The plasmids used for transfection were prepared using a plasmid midi kit ([Qiagen](http://www.jbc.org/cgi/redirect-inline?ad=Qiagen), Venlo, Netherlands). Consistency between plasmid preparations was monitored by determining their concentrations by spectrophotometry and agarose gel electrophoresis.

**Fluorescence resonance energy transfer (FRET)**

For FRET analysis, YFP-SERT (Y-SERT) and CFP-Flot1 (C-Flot1) were co-transfected in HEK293 cells using the calcium phosphate co-precipitation method, as described elsewhere (Sucic *et al.* 2010). A SERT construct tagged with CFP and YFP on its cytoplasmic N and C termini, respectively (to yield C-SERT-Y; (Just *et al.* 2004)), was used as a positive control; CFP-SERT and YFP-myrpalm served as negative controls for FRET experiments.

The ‘three-filter method’ was performed as previously described (Bartholomäus *et al.* 2008). Images were acquired using a 63× oil immersion objective under continuous usage of a grey filter (20% density). LUDL filter wheels allowed for a rapid excitation and emission filter exchange. The LUDL filter wheels were configured as follows: CFP (*I*_Donor_; excitation: 436 nm, emission: 480 nm, and dichroic mirror: 455 nm), YFP (*I*_Acceptor_; excitation: 500 nm, emission: 535 nm, and dichroic mirror: 515 nm) and FRET (I_FRET_; excitation: 436 nm, emission: 535 nm, and dichroic mirror: 455 nm). Images were acquired with a CCD camera (Coolsnap *fx*, Roper Scientific) using the MetaMorph of MetaSeries software package (release 4.6; Universal Imaging Corp., Downing-town, PA). Pixelshift was corrected whenever necessary by using the following combination of ImageJ-plugins: TurboReg and StackReg (Thévenaz *et al.* 1998). Background fluorescence was subtracted from all images. We analyzed the images pixel by pixel using ImageJ (Wayne Rassband, National Institute of Health, version 1.43b) and the ImageJ plugin PixFRET (Pixel by Pixel analysis of FRET with ImageJ, version 1.6.0_10; (Feige *et al.* 2005)), and spectral bleed-through (SBT) parameters were determined for the donor bleed through (BT) and the acceptor BT. Next, FRET efficiency (*E*) was computed: the mean FRET efficiency was measured at the plasma membrane (pre-defined as the *region of interest*) using the computed FRET efficiency image. The regions of interest were selected in the CFP (donor) or YFP (acceptor) image (to avoid bleaching-associated bias) and transmitted to the FRET image (equivalent to the Youvan-image, FRETc; (Youvan *et al.* 2002)) by the ImageJ Multi Measure Tool. All experiments were conducted for individual transfections; 5 to 7 wide-field images were captured during each experiment and 1 to 7 transfected cells per image were included in the study. Distances *r* were calculated based on the Förster equation using the value of 4.92 nm as *R*_0_ for the CFP-YFP FRET pair according Patterson *et al.* (Patterson *et al.* 2000):

$E= \frac{R_{0}^{6}}{(R_{0}^{6}{+ r}^{6})}$.

**Quantitative real-time PCR**

mRNA extraction from manually dissected hippocampus or dorsal raphe nucleus (DRN) tissue was performed using the miRNeasy Mini Kit (Qiagen, Venlo, Netherlands) according to the manufacturer’s protocol. 900 ng of total RNA were used in the subsequent cDNA synthesis step with the DyNAmo cDNA Synthesis Kit (ThermoScientiﬁc, Waltham, MA, USA).

For qRT-PCR, 7.5 µL SYBR Green MasterMix (LifeTechnologies, Carlsbad, CA, USA) and 0.15 µL each of forward and reverse primers (for primer sequences, see Table 1), as well as 6.2 µL of RNase-free water and 1µL of diluted sample were combined for each reaction repeat in a 96-well qRT-PCR plate. All reactions were carried out in duplicates. C(t) cycle values were determined for the target gene and the housekeeping gene β-actin, which were subtracted for each sample to obtain ΔC(t), corresponding to the relative quantification of target mRNA in each sample. ΔΔC(t) values were then calculated by subtracting the mean WT/baseline ΔC(t) from each sample’s ΔC(t). Using the formula 2 ^-ΔΔC(t)^ then allowed the expression of the results in terms of mean fold change of target mRNA levels between experimental groups and the WT/baseline control group (here 2 ^-ΔΔC(t)^=1).

**Electrophysiological recordings**

Mice were deeply anesthetized with a ketamine/xylazine cocktail (Ketanest, 100 mg/kg, Pfizermed, New York, NY, USA; Rompun, 20mg/kg; Bayer, Leverkusen, Germany; 10 mL/kg intraperitoneally) and placed into a stereotaxic frame (Kopf Instruments, Tujunga, CA, USA). Electrophysiological recordings were performed only after deep anesthesia was confirmed in each animal by testing the limb retraction reflex.

Standard borosilicate-glass pipettes, obtained by using a horizontal micropipette puller (Sutter Instrument, Novato, CA, USA) and designed to exhibit resistances of 5–7 MΩ once filled with artificial cerebrospinal fluid (aCSF: 125 mM NaCl, 2,5 mM KCl, 1,25 mM NaH_2_PO_4_, 25 mM D-(+)Glucose, 25 mM NaHCO_3_, 1 mM MgCl_2_, 2 mM CaCl_2_, pH=7.4).

To locate the dorsal raphe nucleus for recordings, a correction factor was first calculated for each mouse to account for individual differences in skull anatomy, by relating the measured distance between two important landmarks to that given by a standard Mouse Brain Atlas (Paxinos 2013) according to the following formula (Athos & Storm 2001):

$$f=\frac{\left( Measured Distance Bregma-Lambda \right)}{(Distance Bregma-Lambda in Atlas)}$$

This factor was then applied to the rostrocaudal coordinates of the DRN given by a standard mouse brain atlas (Paxinos 2013): -4.5mm Bregma.

The recording electrodes were lowered in 0.5 mm-steps until identification of the DRN according to several characteristics previously described to distinguish the firing pattern of serotonergic neurons: Regular (0.5-2.5 Hz) relatively long-duration spikes (0.8-1.2 ms) (Gobbi *et al.* 2001; Lira *et al.* 2003). Upon positive identification of serotonergic spikes, multiple dorsoventral locations were recorded for at least 4 minutes each, and the average firing frequency was calculated for both WT and KO mice as the number of events over recorded bursting times.

Extracellular recordings were obtained using an AxoClamp-2B amplifier (Axon Instruments, Union City, CA, USA) in the bridge mode configuration. Analogue data was digitized via an Axon Instruments Digidata-1440 interface and processed using the pClamp-10 software package (Axon Instruments, Union City, CA, USA ). Analysis of the recordings was performed using Clampfit (Axon Instruments, Union City, CA, USA ) and its Threshold Search option, enabling the upper noise limit to be set as a threshold (typically 50-100 µV).

**Chronic corticosterone treatment**

Chronic corticosterone (CORT) treatment was modified from David *et al.* (David *et al.* 2009). Briefly, mice were single-housed for 3-5 days prior to the start of CORT treatment. A 35 μg/mL CORT solution (100% dosage) was prepared by dissolving CORT (Sigma Aldrich, St. Louis, MO, USA) in a 0.45% β-cyclodextrin solution (Sigma Aldrich, St. Louis, MO, USA) in tap water. The solution was filled into opaque drinking bottles and used to replace the regular drinking water in the cage. The solution was freshly prepared every 72 hours. Mice were exposed to the CORT solution for 3 weeks, followed by a weaning phase of six days (3 days at 50% dosage, 3 days at 25% dosage) and a wash-out period of 2 weeks (Gourley & Taylor 2009) after which all experiments were conducted.

**Immunohistochemistry**

Mice were transcardially perfused with 0.9% saline solution and 4% paraformaldehyde after terminal anesthesia using ketamine-xylazine (100 mg/kg and 40mg/kg respectively; 10 mL/kg i.p.). Brains were extracted and frozen at -80°C in a 70-30 mixture of Tissue-Tek O.C.T. Compound (Sakura, Alphen aan den Rijn, Netherlands) and 30% sucrose. Using the Mouse Brain Atlas for reference (Paxinos 2013), slices containing the dorsal raphe nucleus were cut (30 μm) on a cryostat (Leica cm1950, Leica, Wetzlar, Germany). Slices were stored in cryoprotective solution (30% glycerol, 30% ethylene, 40% PBS) at -20°C until further use.

For immunohistochemistry, slices were washed three times for 10 minutes in TBS (0.1M Tris in 0.9% NaCl, pH=7.4) before shaking in 200 μL blocking solution (5% donkey serum in TBS, 0.3% Triton-X) for 30 minutes at room temperature. Next, slices were incubated for 72 hours at 4°C on a shaker with the primary anti-5-HT antibody (1:200, ab66047, Abcam, Cambridge, UK). After three 15-min washes, slices were incubated with the secondary antibody (Alexa Fluor® Donkey anti-goat IgG, 200 μL per well of 1:200 in TBS with 10% donkey serum, A11055, Life technologies Corp., Eugene, OR, USA) for 1.5 hours at room temperature. After three more 15-minute washes with TBS, slices were mounted onto glass slides (SuperFrost Plus, VWR, Radnor, PA, USA) with Fluorogel (Science Services, Munich, Germany). Slides were left to dry and then imaged using a fluorescence microscope (Axiovert 200M, Zeiss, Oberkochen, Germany) at 5× and 10× magnification.

Alexa Fluor-positive cells were manually counted by an experimenter blinded to the experimental conditions using ImageJ software (NIH, Bethesda, MA, USA). Four sections from each animal, corresponding to the caudal DRN (-4,96 mm Bregma), the middle DRN (two sections, -4,78 and -4,60 mm Bregma respectively), and the rostral DRN (-4,48 Bregma), were used in the counting procedure. Regions of interest were defined according to the anatomical atlases of the dorsal raphe nucleus published by Abrams *et al.* (Abrams *et al.* 2004).

**Statistics**

Data were tested for normality using the D’Agostino-Pearson omnibus test prior to further statistical evaluation. For statistical analyses of differences between two groups, unpaired two-tailed Student’s t tests or, for data sets with unequal variances, Welch’s t tests were employed. For more than two groups, a one-way ANOVA was performed. For experiments involving two groups and two conditions (2 x 2 design), two-way ANOVA was carried out. In both cases, Tukey’s post hoc test with adjusted *p* values to account for multiple comparisons was used where indicated. An α-level of 0.05 was adopted in all instances. All analyses were carried out using Graphpad Prism 7 statistical analysis program (Graphpad Software, La Jolla, CA, USA).

**Supplemental References**

Abrams, J.K., Johnson, P.L., Hollis, J.H. & Lowry, C.A. (2004) Anatomic and functional topography of the dorsal raphe nucleus. *Ann N Y Acad Sci* **1018**, 46–57.

Athos, J. & Storm, D.R. (2001) High precision stereotaxic surgery in mice. *Curr Protoc Neurosci* **Appendix 4**, Appendix 4A.

Bartholomäus, I., Milan-Lobo, L., Nicke, A., Dutertre, S., Hastrup, H., Jha, A., Gether, U., Sitte, H.H., Betz, H. & Eulenburg, V. (2008) Glycine Transporter Dimers. *J Biol Chem* **283**, 10978–10991.

David, D.J., Samuels, B.A., Rainer, Q., Wang, J.-W., Marsteller, D., Mendez, I., Drew, M., Craig, D.A., Guiard, B.P., Guilloux, J.-P., Artymyshyn, R.P., Gardier, A.M., Gerald, C., Antonijevic, I.A., Leonardo, E.D. & Hen, R. (2009) Neurogenesis-Dependent and -Independent Effects of Fluoxetine in an Animal Model of Anxiety/Depression. *Neuron* **62**, 479–493.

Feige, J.N., Sage, D., Wahli, W., Desvergne, B. & Gelman, L. (2005) PixFRET, an ImageJ plug-in for FRET calculation that can accommodate variations in spectral bleed-throughs. *Microsc Res Tech* **68**, 51–58.

Glebov, O.O., Bright, N.A. & Nichols, B.J. (2006) Flotillin-1 defines a clathrin-independent endocytic pathway in mammalian cells. *Nat Cell Biol* **8**, 46–54.

Gobbi, G., Murphy, D.L., Lesch, K. & Blier, P. (2001) Modifications of the serotonergic system in mice lacking serotonin transporters: an in vivo electrophysiological study. *J Pharmacol Exp Ther* **296**, 987–995.

Gourley, S.L. & Taylor, J.R. (2009) Recapitulation and reversal of a persistent depression-like syndrome in rodents. *Curr Protoc Neurosci* **Chapter 9**, Unit 9.32.

Griesauer, I., Diao, W., Ronovsky, M., Elbau, I., Sartori, S., Singewald, N. & Pollak, D.D. (2014) Circadian abnormalities in a mouse model of high trait anxiety and depression. *Ann Med* **46**, 148–154.

Just, H., Sitte, H.H., Schmid, J.A., Freissmuth, M. & Kudlacek, O. (2004) Identification of an additional interaction domain in transmembrane domains 11 and 12 that supports oligomer formation in the human serotonin transporter. *J Biol Chem* **279**, 6650–6657.

Khan, D., Fernando, P., Cicvaric, A., Berger, A., Pollak, A., Monje, F.J. & Pollak, D.D. (2014) Long-term effects of maternal immune activation on depression-like behavior in the mouse. *Transl Psychiatry* **4**, e363.

Lira, A., Zhou, M., Castanon, N., Ansorge, M.S., Gordon, J.A., Francis, J.H., Bradley-Moore, M., Lira, J., Underwood, M.D., Arango, V., Kung, H.F., Hofer, M.A., Hen, R. & Gingrich, J.A. (2003) Altered depression-related behaviors and functional changes in the dorsal raphe nucleus of serotonin transporter-deficient mice. *Biol Psychiatry* **54**, 960–971.

Ludwig, A., Otto, G.P., Riento, K., Hams, E., Fallon, P.G. & Nichols, B.J. (2010) Flotillin microdomains interact with the cortical cytoskeleton to control uropod formation and neutrophil recruitment. *J Cell Biol* **191**, 771–781.

Monje, F.J., Cabatic, M., Divisch, I., Kim, E.-J., Herkner, K.R., Binder, B.R. & Pollak, D.D. (2011) Constant darkness induces IL-6-dependent depression-like behavior through the NF-κB signaling pathway. *J Neurosci* **31**, 9075–9083.

Patterson, G.H., Piston, D.W. & Barisas, B.G. (2000) Förster distances between green fluorescent protein pairs. *Anal Biochem* **284**, 438–440.

Paxinos, G. (2013) *Paxinos and Franklin’s the mouse brain in stereotaxic coordinates /*. 4th ed. Boston :, Amsterdam :

Savalli, G., Diao, W., Berger, S., Ronovsky, M., Partonen, T. & Pollak, D.D. (2015) Anhedonic behavior in cryptochrome 2-deficient mice is paralleled by altered diurnal patterns of amygdala gene expression. *Amino Acids* **47**, 1367–1377.

Schuck, S., Honsho, M., Ekroos, K., Shevchenko, A. & Simons, K. (2003) Resistance of cell membranes to different detergents. *PNAS* **100**, 5795–5800.

Sucic, S., Dallinger, S., Zdrazil, B., Weissensteiner, R., Jørgensen, T.N., Holy, M., Kudlacek, O., Seidel, S., Cha, J.H., Gether, U., Newman, A.H., Ecker, G.F., Freissmuth, M. & Sitte, H.H. (2010) The N Terminus of Monoamine Transporters Is a Lever Required for the Action of Amphetamines. *J Biol Chem* **285**, 10924–10938.

Thévenaz, P., Ruttimann, U.E. & Unser, M. (1998) A pyramid approach to subpixel registration based on intensity. *IEEE Trans Image Process* **7**, 27–41.

Youvan, D.C., Silva, C.M., Bylina, E.J., Coleman, W.J., Dilworth, M.R. & Yang, M.M. (2002) Calibration of fluorescence resonance energy transfer in microscopy.

Zhuang, X., Masson, J., Gingrich, J.A., Rayport, S. & Hen, R. (2005) Targeted gene expression in dopamine and serotonin neurons of the mouse brain. *J Neurosci Methods* **143**, 27–32.

**Supplementary Figure Legends**

**Figure 1. Analysis of hippocampal glucocorticoid receptor protein levels by subcellular compartment in response to chronic corticosterone in Flot1 KO and WT mice.**

Chronic CORT treatment results in a significant reduction in relative GR protein in the **(a)** cytosol (genotype: *F*_(1,20)_=2.020, p=0.171; treatment: *F*_(1,20)_=9.805, p=0.005; genotype × treatment: *F*_(1,20)_ =1.043, p=0.319; n=6/group) and **(b)** the nucleus (genotype: *F*_(1,21)_=1.041, p=0.319; treatment: *F*_(1,21)_=5.699, p=0.026; genotype × treatment: *F*_(1,21)_ =4.024, p=0.058; n=4-8/group) in both Flot1 KO and WT mice, which was also reflected in **(c)** total relative GR levels in the hippocampus (genotype: *F*_(1,18)_=3.103, p=0.095; treatment: *F*_(1,18)_=7.778, p=0.0121; genotype × treatment: *F*_(1,18)_ =3.652, p=0.0721; n=4-6/group). Data are depicted as mean ± SEM; main effect of treatment depicted as: ♦ p<0.05 / ♦♦ p<0.01.

**Figure 2. Timeline displaying the sequence of behavioral testing.**

A 24 hour time interval was observed between the individual behavioral tests which were carried out in order or increasing stressfulness.
