# Supplementary figures and images for: Flotillin‐1 interacts with the serotonin transporter and modulates chronic corticosterone response
Source: Genes Brain Behav. 2018 May 20;18(2):e12482. doi: 10.1111/gbb.12482 (PMC6392109; doi:10.1111/gbb.12482)

# Supplementary Figure 1

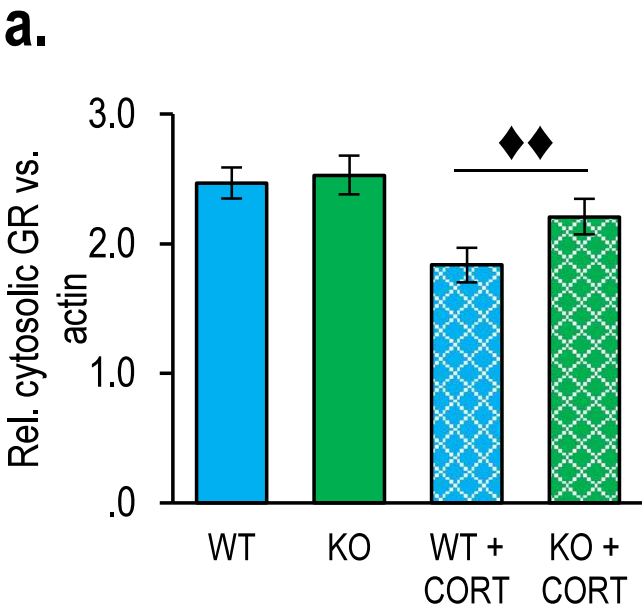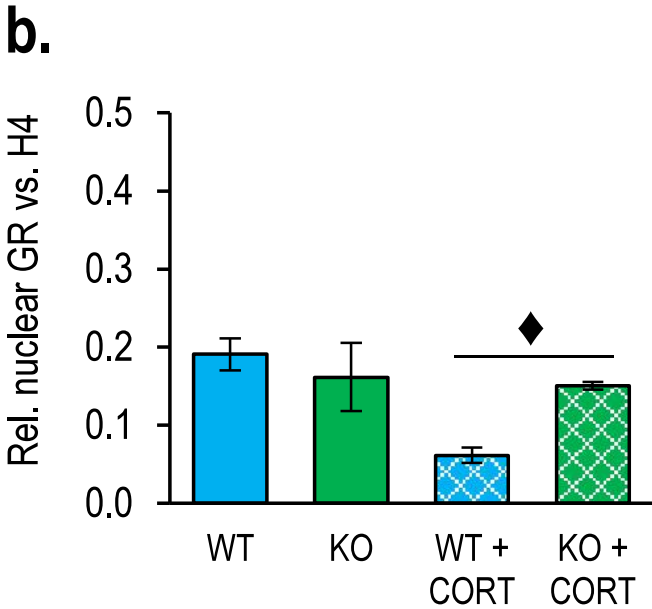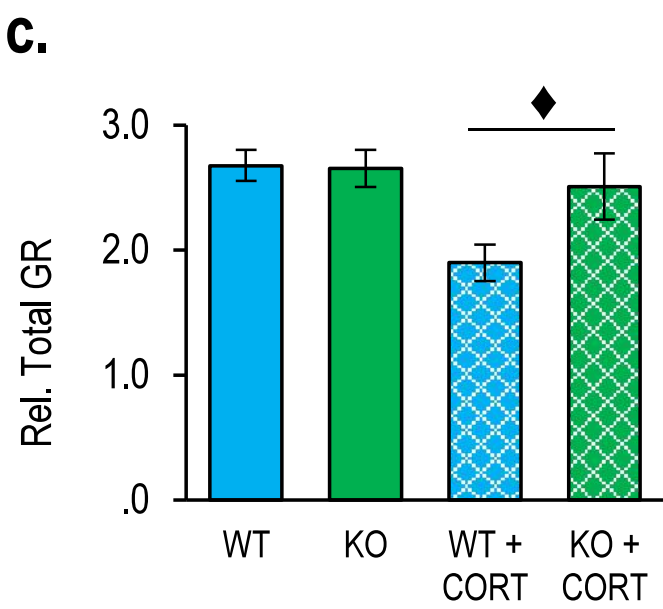

Supplement: Supplementary file 2 — Figure S1. Analysis of hippocampal glucocorticoid receptor protein levels by subcellular compartment in response to chronic corticosterone in Flot1 KO and WT mice. Chronic CORT treatment results in a significant reduction in relative GR protein in the (A) cytosol (genotype: F 1,20 = 2.020, P = .171; treatment: F 1,20 = 9.805, P = .005; genotype × treatment: F 1,20 = 1.043, P = .319; n = 6/group) and (B) the nucleus (genotype: F 1,21 = 1.041, P = .319; treatment: F 1,21 = 5.699, P = .026; genotype × treatment: F 1,21 = 4.024, P = .058; n = 4‐8/group) in both Flot1 KO and WT mice, which was also reflected in (C) total relative GR levels in the hippocampus (genotype: F 1,18 = 3.103, P = .095; treatment: F 1,18 = 7.778, P = .0121; genotype × treatment: F 1,18 = 3.652, P = .0721; n = 4‐6/group). Data are depicted as mean ±SEM; main effect of treatment depicted as: ♦ P < .05; ♦♦ P < .01 [file GBB-18-na-s003.pdf]
