# Supplementary material for: Flotillin‐1 interacts with the serotonin transporter and modulates chronic corticosterone response
Source: Genes Brain Behav. 2018 May 20;18(2):e12482. doi: 10.1111/gbb.12482 (PMC6392109; doi:10.1111/gbb.12482)

## Supplementary Figure 2

Timeline of behavior experiments (at least 24h between tests)

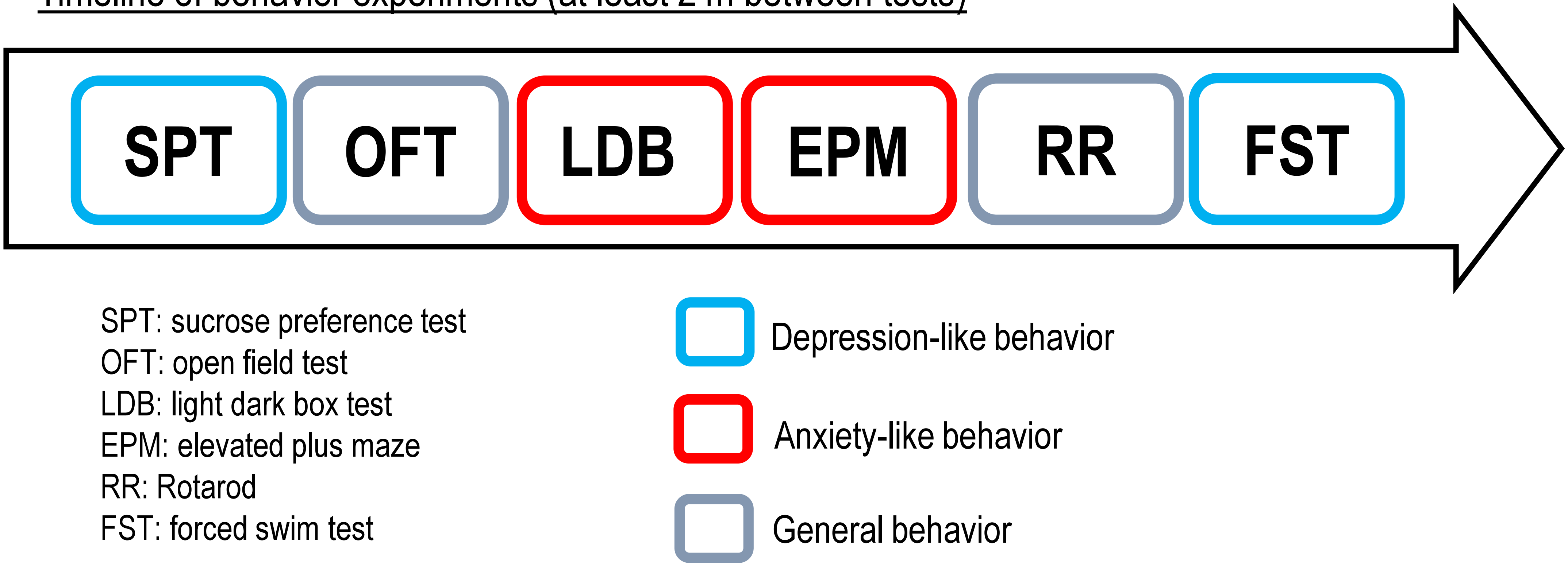

Supplement: Supplementary file 3 — Figure S2. Timeline displaying the sequence of behavioral testing. A 24 hour time interval was observed between the individual behavioral tests which were carried out in order or increasing stressfulness [file GBB-18-na-s001.pdf]
